# Oscillations of retaining wall subject to Grob’s swelling pressure

**DOI:** 10.1038/s41598-022-15591-y

**Published:** 2022-07-18

**Authors:** Maksim Kozlov, Aizhan Tulendinova, Jong Kim, Grant Ellis, Piotr Skrzypacz

**Affiliations:** 1 Center for Preparatory Studies, Nazarbayev University, 53 Kabanbay Batyr Ave, Nur-Sultan, 010000 Kazakhstan; 2 Department of Mathematics, School of Sciences and Humanities, Nazarbayev University, 53 Kabanbay Batyr Ave, Nur-Sultan, 010000 Kazakhstan; 3 Department of Civil and Environmental Engineering, School of Engineering and Digital Sciences, Nazarbayev University, 53 Kabanbay Batyr Ave, Nur-Sultan, 010000 Kazakhstan; 4 Unaffiliated, Seattle, USA

**Keywords:** Engineering, Civil engineering

## Abstract

The single-degree-of-freedom nonlinear problem describing the essential dynamics of an oscillating retaining wall based on non-quaking ground and subject to Grob’s swelling pressure is considered. The periodic solutions are derived using harmonic approximation. The amplitude-frequency relation is established by employing Lambert’s special function or alternatively using linearization of the nonlinear force. Analytical results are verified using numerical simulations.

## Introduction

Retaining walls and foundations in construction are often subject to the swelling pressure caused by expansive soils such as clay or soft rock. This pressure can result in significant vibrations of structures and hence lead to damage and economical loss. Predicting the effect of swelling pressure on structures is therefore an important problem in mechanical and civil engineering, see^1–4^. Vibration analysis of retaining walls is useful in many building and construction applications. These include building vibrations induced by high-speed trains moving on bridges^5^, seismic analysis in and around earthquake zones^6^, and vibrations incurred in construction sites^7,8^. This analysis is critical for retaining wall structures around power plants especially nuclear. The Fukushima nuclear disaster in 2011 was a result of the Tōhoku earthquake and tsunami.

Vibrations of retaining walls caused by dynamic (seismic) loading were investigated analytically using linear approximation^9^. Experimental and numerical results for concrete retaining walls under low-frequency dynamic loading were reported in^10^. In this work, we present an analysis of the lumped mass model for retaining walls subject to the swelling pressure that obeys Grob's semi-logarithmic law for the volumetric stress^1^. The periodic solutions are derived using harmonic approximation. The amplitude-frequency relation is established by employing Lambert’s special function or using linearization of the nonlinear force term. Analytical results are verified by numerical simulations. The approximations that we derive can be used to evaluate the frequency and amplitude of oscillations without time-consuming finite element calculations.

This paper is organized as follows. The mathematical model for retaining walls subject to the swelling pressure, the one-mode Galerkin approximation, and analytical results for the nonlinear conservative oscillator are presented in “Mathematical model for retaining wall subject to swelling pressure”. In “Linear approximation”, we study the linear model, and in “Harmonic approximations for nonlinear oscillator”, we apply harmonic ansatz to the nonlinear lumped mass model and derive closed-form formulas for the approximate frequency and amplitude of oscillations of the retaining wall. In “Comparison of approximation methods”, the effects of parameters on amplitude and frequency of oscillations are studied and various approximation methods are verified against a numerical solution. Finally, conclusions are drawn in “Conclusions”.

## Mathematical model for retaining wall subject to swelling pressure

### Cantilever beam subject to Grob’s swelling pressure

**Figure 1:**
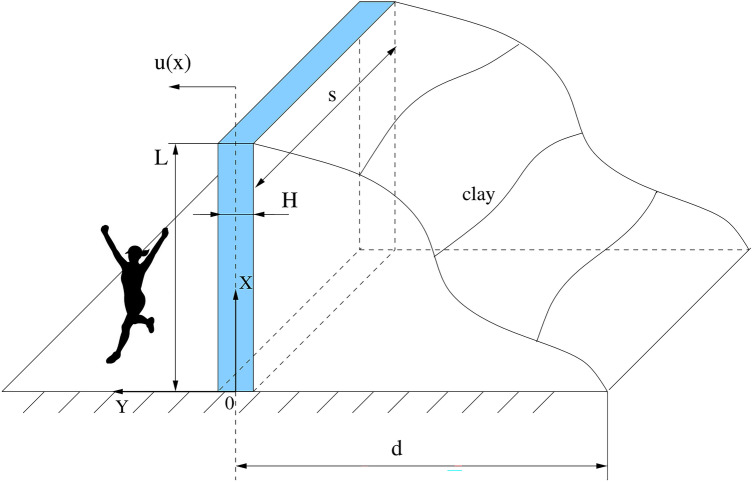
Retaining wall based on the non-quaking ground.

Swelled soil exerts pressure (stress) on the retaining wall. This pressure reaches its maximum at the original undeformed position of the wall which corresponds to the maximum of soil compression. Deflection of the wall from its original position results in expansion of soil and therefore a reduction of pressure that should asymptotically approach zero with any further increase of wall deflection (decrease of soil compression). A simple model satisfying this property that was confirmed by a series of experiments, including the combined swell-swell heave, the multi-stepped, and Hunder-Amberg swell tests^11,12^, is the exponential decay

1 $${\sigma }_{s}={\sigma }_{0}{e}^{-Bu},$$

where *u* denotes the deflection of the retaining wall from its axial position, ${\sigma }_{s}$ is the present axial stress and ${\sigma }_{0}$ is the maximal stress for which swelling occurs (equilibrium stress with respect to swelling). The decay rate is given by

2 $$B=\frac{\mathrm{ln}(10)}{cd},$$

where *d* is the thickness of swelled soil layer (compression is equal to *u/d*) and *c* is the experimentally fitted swelling parameter (see Fig. 1). Transverse deformations are not allowed in the abovementioned swelling tests. Eq. (1) is better known in the literature as Grob’s swelling law describing the logarithmic dependence of deformation on pressure^1^

$$\frac{u}{d}=-c\cdot\mathrm{ lo}{\mathrm{g}}_{10}\left(\frac{{\sigma }_{s}}{{\sigma }_{0}}\right).$$

This equation is well accepted in civil engineering areas and many applications can be found in the literature^2,3,13,14^.

Next, a small horizontal deflection of the retaining wall of length *s* can be described by the Euler–Bernoulli elastic beam equation subject to initial/boundary conditions

3 $$\left\{\begin{array}{l}\rho {A}_{b}{u}_{tt}+EI{u}_{xxxx}=s{\sigma }_{0}{e}^{-Bu}, x\in \left(0,L\right),\, t\in (0,{t}_{end}),\\ u\left(t,0\right)={u}_{x}\left(t,0\right)={u}_{xx}\left(t,L\right)={u}_{xxx}\left(t,L\right)=0, \,\,t\in [0,{t}_{end}],\\ u\left(0,x\right)={u}_{0}\left(x\right),\,\,{ u}_{t}\left(0,x\right)=0, \,\,x\in (0,L),\end{array}\right.$$

where $x\in \left[0,L\right]$ is the axial position and *L* is the height of the retaining wall, $E$ and $\rho$ are the modulus of elasticity and the density of the wall material, respectively. ${A}_{b}=sH$ is the area of constant cross-section of rectangular cantilever and its moment of inertia is given by $I=\frac{s{H}^{3}}{12}$.

Notice that the boundary conditions correspond to the case of the cantilever beam whose base is clamped or fixed whereas the top end is free. The first equation in (Eq. 3) corresponds to the second Newton's law of motion. The retaining wall vibrates due to its restoring elastic force and the force resulting from the swelling and contracting clay.

Let us introduce the dimensionless variables

4 $$\widetilde{x}=\frac{x}{L}, \,\,\widetilde{u}=\frac{u}{|{u}_{c}|},\,\, \widetilde{t}=\sqrt{\frac{s{\sigma }_{0}}{|{u}_{c}|\rho {A}_{b}}}t,$$

and define

5 $$\alpha =\frac{|{u}_{c}|EI}{{L}^{4}s{\sigma }_{0}}\quad \mathrm{ and }\quad \beta =B|{u}_{c}|,$$

where ${u}_{c}$ is some predefined characteristic deflection. Then, Eq. (3) can be written as

6 $$\left\{\begin{array}{l}{u}_{tt}+\alpha {u}_{xxxx}={e}^{-\beta u}, \,\,x\in \left(0,1\right),\,\, t\in (0,{t}_{end}),\\ u\left(t,0\right)={u}_{x}\left(t,0\right)={u}_{xx}\left(t,1\right)={u}_{xxx}\left(t,1\right)=0,\,\, t\in [0,{t}_{end}],\\ u\left(0,x\right)={u}_{0}\left(x\right),\,\,{ u}_{t}\left(0,x\right)=0, \,\, x\in (0,1),\end{array}\right.$$

where the tilde notation is omitted for brevity.

### Mass lumped model

Let us assume that the initial/boundary value problem given by Eq. (6) has solution of the form

$$u\left(t,x\right)=\sum_{j=1}^{\infty }{X}_{j}\left(t\right){Y}_{j}\left(x\right),$$

where ${Y}_{j}\left(x\right)$ are eigenmodes of the differential operator $\frac{{d}^{4}}{d{x}^{4}}$ subject to the boundary conditions listed. To study the essential dynamics of the wall subject to the swelling pressure, we employ a single-mode Galerkin approximation

7 $$u\left(t,x\right)\approx X(t)\cdot Y(x),$$

where $Y\left(x\right)$ represents the first eigenmode of the cantilever beam whose base end is clamped, whereas the top end is free. Neglecting the higher frequency modes can yield simple yet accurate approximate solutions to many engineering problems^15^.

The scaled first eigenfunction (eigenmode) of the beam differential operator $\frac{{d}^{4}}{d{x}^{4}}$ subject to the boundary conditions

$$Y(0)={Y}_{x}(0)={Y}_{xx}(1)={Y}_{xxx}(1)=0$$

is given by

8 $$Y(x)=\frac{1}{2}\left({Y}_{3}\left(x,{\mu }_{1}\right)-\frac{{Y}_{1}\left(1,{\mu }_{1}\right)}{{Y}_{2}\left(1,{\mu }_{1}\right)}\cdot {Y}_{4}\left(x,{\mu }_{1}\right)\right),$$

where

$${Y}_{1}\left(x,{\mu }_{1}\right)=\mathrm{cosh}\left({\mu }_{1}x\right)+\mathrm{cos}\left({\mu }_{1}x\right),\,\, {Y}_{2}\left(x,{\mu }_{1}\right)=\mathrm{sinh}\left({\mu }_{1}x\right)+\mathrm{sin}\left({\mu }_{1}x\right),$$

9 $${Y}_{3}\left(x,{\mu }_{1}\right)=\mathrm{cosh}\left({\mu }_{1}x\right)-\mathrm{cos}\left({\mu }_{1}x\right),\,\, {Y}_{4}\left(x,{\mu }_{1}\right)=\mathrm{sinh}\left({\mu }_{1}x\right)-\mathrm{sin}\left({\mu }_{1}x\right),$$

denote Krylov's eigenfunction for the fourth order differential operator $\frac{{d}^{4}}{d{x}^{4}}$ subject to the boundary conditions for the cantilever beam^4,16^. The spectral parameter

${\mu }_{1}=1.8751040687...$ is the first positive root of the transcendental equation

10 $$1+\mathrm{cosh}(\mu )\cdot \mathrm{cos}(\mu )=0.$$

The scaled eigenfunction $Y(x)$ in Eq. (8) has the following properties^4^

11 $$Y\left(1\right)=1,$$

12 $${\int }_{0}^{1}{Y}^{2}\left(x\right)dx=\frac{1}{4},$$

13 $${\int }_{0}^{1}({Y}^{\mathrm{^{\prime}}\mathrm{^{\prime}}}(x){)}^{2}dx={\mu }_{1}^{4}{\int }_{0}^{1}{Y}^{2}(x)dx,$$

and

14 $$Y\left(x\right)>0\quad \mathrm{for }\,\,0<x\le 1.$$

The corresponding Galerkin equation for the retaining wall model based on the non-quaking ground becomes

15 $$\ddot{X}(t){\int }_{0}^{1}{Y}^{2}(x)dx+\alpha X(t){\int }_{0}^{1}({Y}^{\mathrm{^{\prime}}\mathrm{^{\prime}}}(x){)}^{2}dx={\int }_{0}^{1}Y(x){e}^{-\beta X(t)Y(x)}dx.$$

Applying the trapezoidal rule to the integral on the right-hand side of Eq. (15) and using Eqs.(11)– (14) yields

16 $$\ddot{X}\left(t\right)+\alpha {\mu }_{1}^{4}X\left(t\right)=2{e}^{-\beta X(t)}.$$

Substituting

$$\widetilde{X}\left(\widetilde{t}\right)=\beta X\left(\widetilde{t}\right)\approx \frac{\mathrm{ln}\left(10\right)}{cd}u\left(\widetilde{t},L\right)\quad \mathrm{ and }\quad \widetilde{t}=\sqrt{\alpha }{\mu }_{1}^{2}t={\mu }_{1}^{2}\sqrt{\frac{EI}{\rho {A}_{b}{L}^{4}}}t$$

leads to the conservative single-degree-of-freedom oscillator equation in the dimensionless form

17 $$\ddot{X}\left(t\right)+X\left(t\right)=p{e}^{-X\left(t\right)},$$

where the tilde notation was dropped again for the sake of brevity, and dimensionless parameter *p* is defined as

18 $$p=\frac{2\beta }{{\mu }_{1}^{4}\alpha }=\frac{2{L}^{4}s{\sigma }_{0}B}{{\mu }_{1}^{4}EI}.$$

In this work, the non-linear oscillator equation (17) subject to zero-initial conditions is assumed

19 $$X\left(0\right)=0,\quad \dot{X}\left(0\right)=0.$$

Equations (17) and (19) constitute a zero-initial value problem for a conservative nonlinear oscillator. We expect a bounded periodic solution whose amplitude is a monotonically increasing function of the swelling parameter *p*. For higher values of *p,* the dynamics can be affected by the presence of higher-order harmonics.

Multiplying Eq. (17) by $\dot{X}\left(t\right)$ and integrating with respect to time, we get the conservation of energy

20 $$\frac{1}{2}{\left(\dot{X}(t)\right)}^{2}+\frac{1}{2}{X}^{2}(t)+p{e}^{-X(t)}=C$$

where $C=p$ due to the initial conditions (Eq. 19). Thus,

21 $${\dot{X}(t)}^{2}=2p-{X}^{2}(t)-2p{e}^{-X(t)}.$$

We now show that all solutions to the initial value problem (17) and (19) are periodic.

#### Theorem 1

The initial value problem (17) with initial conditions (19) has a periodic non-negative solution for any positive lumped mass model parameters *p*.

#### Proof

We establish the proof using simple phase plane analysis, cf.,^4,17–19^. The solution $X\left(t\right)$ is periodic if and only if the phase diagram produces a closed curve. This holds true if the continuous function

22 $$g\left(s\right)=2p-{s}^{2}-2p{e}^{-s}$$

has two real roots ${s}_{1,2}$, and $g\left(s\right)>0$ for all *s* between ${s}_{1}$ and ${s}_{2}$. Note that $g\left(0\right)=0$, and $g\left(s\right)$ has only one local maximum due to the fact that ${g}^{\mathrm{^{\prime}}}(s)=2p{e}^{-s}-2s=0$ has only one real root ${s}^{*}$ due to Rolle's theorem, and that $g^{\prime\prime} ({s}^{*})=-2p{e}^{-{s}^{*}}-2<0$ holds true for the positive lumped mass model parameter *p*, i.e., *g*(*s*) is a concave function on the whole real line. Hence, the existence of the second root follows from the Intermediate Value Theorem. Now, if we set ${s}^{*}={X}_{eq}$, the following condition is satisfied $p{e}^{-{X}_{eq}}={X}_{eq}$ whence it follows that the solution to the lumped mass model is a constant $X(t)={X}_{eq}$ representing the stable steady state of Eq. (17). To show that the periodic solution $X\left(t\right)$ is non-negative, we rewrite Eq. (21) as follows

23 $${\dot{X}(t)}^{2}+{X}^{2}(t)=2p-2p{e}^{-X(t)}.$$

Since the left-hand side of Eq. (23) is non-negative, it must hold true that $X\left(t\right)\ge 0.$ **∎**

Integrating Eq. (23), we obtain for $0\le t\le T/2$ the exact solution in the implicit form

24 $${\int }_{0}^{X\left(t\right)}\frac{ds}{\sqrt{2p-{s}^{2}-2p{e}^{-s}}}=t.$$

It follows from Eq. (23) that ${X}^{2}(t)\le 2p\left(1-{e}^{-X(t)}\right)$ and consequently

25 $$X\left(t\right)\le \sqrt{2p}{\left(1-{e}^{-\sqrt{2p}}\right)}^{1/2}.$$

Therefore, the maximum deflection *A* satisfies

26 $$A\le \sqrt{2p}{\left(1-{e}^{-\sqrt{2p}}\right)}^{1/2}\le \sqrt{2p}.$$

Notice that the exact value of the maximum deflection is represented by the root of the transcendental equation

27 $${A}^{2}=2p-2p{e}^{-A}$$

The Taylor expansion for $A=A\left(p\right)$ is obtained using successive implicit differentiation with respect to *p*:

28 $$A=2p-4{p}^{2}+20{p}^{3}-160{p}^{4}+....$$

It follows from Eq. (27) that

29 $$p=\frac{{A}^{2}}{2\left(1-{e}^{-A}\right)}$$

and

$$\frac{dA}{dp}=\frac{1}{dp/dA}=\frac{4{\left(1-{e}^{-A}\right)}^{2}}{4A-{e}^{-A}\left(4A+2{A}^{2}\right)}\ge 0.$$

Consequently, the maximum deflection *A* increases with increasing parameter *p*.

#### Remark

Equation (27) can be rewritten as

$${e}^{A}\left(A+\sqrt{2p}\right)\left(A-\sqrt{2p}\right)=-2p.$$

The exact solution of the above transcendental equation can be expressed in terms of the generalized Lambert W function $W\left({t}_{1},{t}_{2};z\right)$^20^ as follows

$$A=W\left(-\sqrt{2p},\sqrt{2p};-2p\right).$$

**Figure 2:**
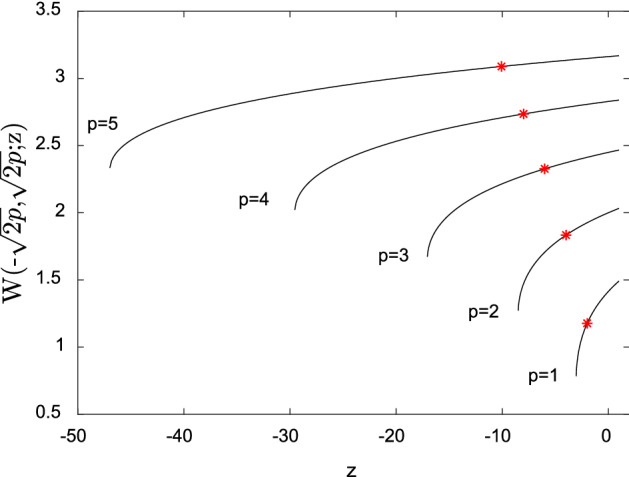
The generalized Lambert W function $W\left(-\sqrt{2p},\sqrt{2p};z\right)$ for parameters p = 1, …, 5. The corresponding solutions $W\left(-\sqrt{2p},\sqrt{2p};-2p\right)$ are indicated by asterisks.

This special function is introduced in^20^ as inverse to the mapping $z\mapsto \left(z-{t}_{1}\right)\left(z-{t}_{2}\right){e}^{z}$. Figure 2 presents the graph of the function $W\left(-\sqrt{2p},\sqrt{2p};z\right)$ for different values of parameter *p*, evaluated by solving ${e}^{W}\left(W+\sqrt{2p}\right)\left(W-\sqrt{2p}\right)=z$ numerically for *W*.

The solution of Eq. (27) is given by the value of $W\left(-\sqrt{2p},\sqrt{2p};z\right)$ at $z=-2p$. The corresponding solutions for various values of parameters *p* are indicated by asterisks. Again, the maximum deflection *A* increases with the increasing value of *p*. □

In the following theorem we estimate the period of oscillation.

#### Theorem 2

The solution to the initial value problem (17) with initial conditions  (19) has a period

$$T= 2\pi \sqrt{\frac{1-{e}^{-A}}{A}}\left(1+\mathcal{O}\left({A}^{2}\right)\right)=2\pi \left(1-\frac{A}{4}+\mathcal{O}\left({A}^{2}\right)\right),$$

where *A* is the maximum deflection.

#### Proof

By Eqs. (24) and (29), the period of the oscillation is given by

30 $$T=2{\int }_{0}^{A}\frac{ds}{\sqrt{2p-{s}^{2}-2p{e}^{-s}}}=2\sqrt{1-{e}^{-A}}{\int }_{0}^{A}\frac{ds}{\sqrt{A-{s}^{2}+{e}^{-A}{s}^{2}-{A}^{2}{e}^{-s}}}=2\sqrt{1-{e}^{-A}}{\int }_{0}^{1}\frac{dt}{\sqrt{1-{t}^{2}+{e}^{-A}{t}^{2}-{e}^{-At}}}.$$

The last integral in Eq. (30) can be approximated as follows

31 $${\int }_{0}^{1}\frac{dt}{\sqrt{1-{t}^{2}+{e}^{-A}{t}^{2}-{e}^{-At}}}=\frac{\pi }{\sqrt{A}}\left(1+\mathcal{O}\left({A}^{2}\right)\right)$$

since

32 $$\frac{1}{\sqrt{1-{t}^{2}+{e}^{-A}{t}^{2}-{e}^{-At}}}=\frac{1+\mathcal{O}\left({A}^{2}\right)}{\sqrt{t\left(1-t\right)A}}$$

for all $0<t<1$ and $A>0$. Therefore,

33 $$T=2\pi \sqrt{\frac{1-{e}^{-A}}{A}}\left(1+\mathcal{O}\left({A}^{2}\right)\right)=2\pi \left(1-\frac{A}{4}+\mathcal{O}\left({A}^{2}\right)\right)$$

due to $$\sqrt{\frac{1-{e}^{-A}}{A}}=1-\frac{A}{4}+\mathcal{O}\left({A}^{2}\right)$$. **∎**

#### Remark

Since $1-{t}^{2}+{e}^{-A}{t}^{2}-{e}^{-At}>t\left(1-t\right)A$ for all $A>0$ and $0<t<1$, one obtains $T>2\pi \sqrt{\frac{1-{e}^{-A}}{A}}$. Computing the exact value of the improper integral in Eq. (31) for $A>0$ remains an open problem. □

## Linear approximation

Now, consider the linear approximation of Eq. (17)

34 $$\ddot{X}\left(t\right)+X\left(t\right)=p\left(1-X\left(t\right)\right).$$

with the corresponding energy conservation

35 $$\frac{1}{2}{\left(\dot{X}\right)}^{2}+\frac{1}{2}{X}^{2}=p\left(X-\frac{{X}^{2}}{2}\right).$$

Substituting $\dot{X}=0$ into the above equation one finds the amplitude of oscillations

36 $$A=\frac{2p}{1+p}=2p-2{p}^{2}+2{p}^{3}-2{p}^{4}+....$$

Note that the approximate amplitude by Eq. (36) coincides with its exact value by Eq. (28) up to the first order with respect to the parameter *p.*

Substituting $\chi =\left(1+1/p\right)X-1$ into Eq. (34) one obtains the equation of harmonic oscillator

37 $$\ddot{\chi }+{\omega }^{2}\chi =0,$$

where the angular frequency is given by

38 $${\omega }^{2}=1+p.$$

## Harmonic approximations for nonlinear oscillator

In the following, we construct approximate periodic solutions to the non-linear oscillator equation (17) subject to zero-initial conditions in Eq. (19) by substituting the harmonic ansatz

39 $$X\left(t\right)\approx \frac{A}{2}\cdot \left(1-\mathrm{cos}\left(\omega t\right)\right)=A{\mathrm{sin}}^{2}\left(\frac{\omega }{2}t\right).$$

The equilibrium position is found by setting $\ddot{X}=0$ in Eq. (17):

40 $${X}_{c}=W\left(p\right),$$

where $W\left(z\right)$ again denotes the Lambert W function^20–24^ which is defined as the inverse of the mapping $z\mapsto z{e}^{z}$, and $W\left(z\right)$ solves the equation

41 $$W\left(z\right){e}^{W\left(z\right)}=z.$$

Notice that the Lambert W function is uniquely defined for $z\ge 0$ and increases monotonically. The Taylor expansion of the Lambert W function is given by

42 $$W\left(z\right)=z-{z}^{2}+\frac{3}{2}{z}^{3}-\frac{8}{3}{z}^{4}+\frac{125}{24}{z}^{5}+O({z}^{6}).$$

The maximum deflection *A* can be found more precisely using the method of successive approximations. Substituting $A_{i+1}=A_i+\Delta A$ into Eq. (27) and neglecting terms that are of second order with respect to $\Delta A$ results in

43 $${A}_{i}^{2}+2\Delta A{A}_{i}=2p\left(1-{e}^{-{A}_{i}}{e}^{-\Delta A}\right).$$

Solving Eq. (43) with respect to $\Delta A$ we obtain recursive relation

44 $${A}_{i+1}=\frac{{A}_{i}}{2}+\frac{p}{{A}_{i}}+W\left(-\frac{\mathrm{exp}\left(-p/{A}_{i}\right)}{2}\right).$$

The initial guess

45 $${A}_{0}=2{X}_{c}=2W\left(p\right)$$

is based on the assumption that the center of the oscillations is close to the equilibrium position (which is a reasonable approximation if oscillations are nearly harmonic).

In particular, the maximum deflection after the first correction is given by

46 $$A=W\left(p\right)+\frac{p}{2W\left(p\right)}+W\left(-\frac{\mathrm{exp}\left(-\frac{p}{2W\left(p\right)}\right)}{2}\right).$$

Following the standard procedure, we approximate the frequency of oscillations by evaluating the derivative of restoring force $f\left(X\right)=X-p{e}^{-X}$ at the equilibrium^25^:

47 $${\omega }^{2}={\left.\frac{df}{dX}\right|}_{X={X}_{c}}=1+W\left(p\right).$$

The above expression for frequency (referred to as the Nayfeh frequency) coincides with frequency of the linear oscillator in Eq. (38) up to first order with respect to *p* while the approximations of the maximum deflection by Eqs. (46) and (36) are identical up to second order with respect to the parameter *p*. Also, the expression for the frequency given by Eq. (47) is consistent with Eq. (33) for the period of oscillations, e.g.,

$$T=\frac{2\pi }{\omega }\approx \frac{2\pi }{\sqrt{1+\frac{A}{2}}}=2\pi \left(1-\frac{A}{4}+\mathcal{O}\left({A}^{2}\right)\right)$$

for $A\approx 2W\left(p\right)$ as shown in Eq. (45).

## Comparison of approximation methods

**Figure 3:**
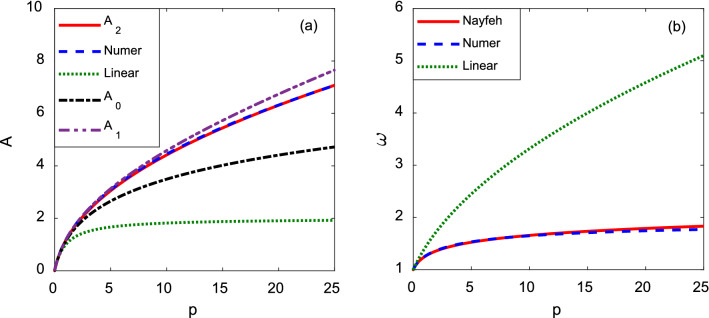
Numerical and approximate analytical values of maximum deflection (**a**) and frequency (**b**) vs. parameter *p***.**

Numerical and approximate values of amplitude and frequency of oscillations vs. parameter *p* are presented in Fig. 3. Figure 3a shows the amplitude *A,* found by direct numerical solution of Eq. (17) (${A}_{numer}$ —blue dashed line), linear approximation given by Eq. (36) (green dotted line), and by using the successive approximations by Eq. (44) (${A}_{0}$—black dash-dotted line dashed line, ${A}_{1}$—purple dash double-dot line and ${A}_{2}$—red solid line).

In Table 1 we list relative errors for the different amplitude approximations

$$\varepsilon =\frac{\left|A-{A}_{num}\right|}{{A}_{num}}$$

for two different values of *p*.

**Table 1:** Relative error for different approximations of amplitude.

*p*	Linear	${A}_{0}$	${A}_{1}$	${A}_{2}$
1.67	$\varepsilon =23\times {10}^{-2}$	$\varepsilon =59\times {10}^{-3}$	$\varepsilon =24\times {10}^{-4}$	$\varepsilon =19\times {10}^{-3}$
25	$\varepsilon =72\times {10}^{-2}$	$\varepsilon =33\times {10}^{-2}$	$\varepsilon =83\times {10}^{-3}$	$\varepsilon =94\times {10}^{-5}$

Figure 3b shows the trend of the frequency $\omega$ vs. parameter *p*, found by direct numerical solution of Eq. (17) (${\omega }_{num}$—blue dashed line), linear approximation given by Eq. (38) (green dotted line), and the Nayfeh approximation given by Eq. (47) (red solid line). In Table 2 we list relative errors for different frequency approximations

$$\delta =\frac{\left|\omega -{\omega }_{num}\right|}{{\omega }_{num}}$$

for two different values of parameter *p*.

**Table 2:** Relative error for different approximations of angular frequency.

*p*	Linear	Nayfeh
1.67	$\delta =22\times {10}^{-2}$	$\delta =5\times {10}^{-3}$
25	$\delta =19\times {10}^{-1}$	$\delta =35\times {10}^{-3}$

**Figure 4:**
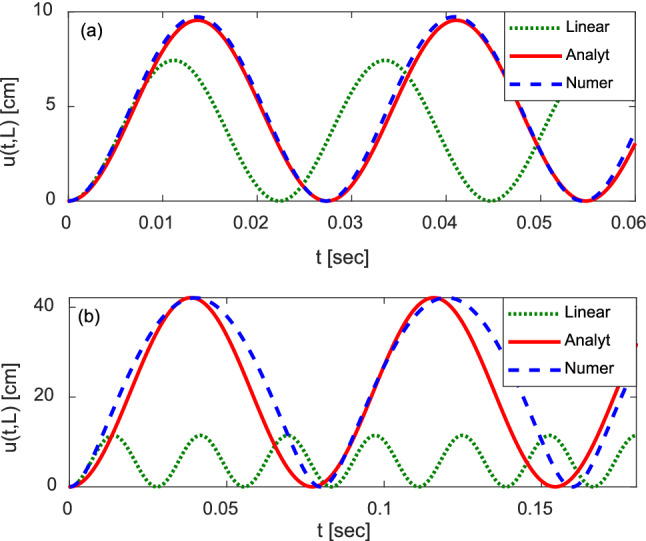
Numerical and approximate analytical solutions for the horizontal displacement of the tip of the cantilever wall $u\left(t,L\right)$ vs. time *t* with (**a**) *L* = 2.54 m and (**b**) *L* = 5 m, d = 4.572 m, E = 20,700 MPa, I = 0.0249739 m, $\rho =2000\mathrm{ kg}/{\mathrm{m}}^{3}$, s = 0.762 m, h = 0.3404 m, c = 0.03, and ${\sigma }_{0}$=1 MPa.

In Fig. 4 we present numerical and approximate analytical solutions for the horizontal deflection of the tip of the cantilever wall $u(t,L)$ vs. time t with the following set of parameters *L* = 2.54 m (Fig. 4a) and *L* = 5 m (Fig. 4b), *d* = 4.572 m, *E* = 20,700 MPa, *I* = 0.0249739 m, $\rho =2000\mathrm{ kg}/{\mathrm{m}}^{3}$, *s* = 0.762 m, *H* = 0.3404 m, *c* = 0.03, and ${\sigma }_{0}$=1 MPa. For this set of parameters, the values of the dimensionless parameter *p* are 1.6663 and 25 for *L* = 2.54 m and *L* = 5 m, respectively. The numerical solution is represented by the blue dashed line. The red solid line corresponds to the harmonic solution given by Eq. (39) with the recursively found maximum deflection ${A}_{2}$ (Eq. 44) and frequency $\omega$ found by Nayfeh approximation [Eq. (47)]. The solution to the linear model [Eq. (38) for frequency and Eq. (36) for the maximum deflection] is represented by green dotted lines.

Observe that while the approximation of amplitude ${A}_{2}$ can be found recursively and remains accurate even in the highly nonlinear regime shown in Fig. 4b, the Nayfeh approximation of frequency or period is not very precise because higher harmonics significantly affect dynamics in this regime.

## Conclusions

This paper presented a simple approach to obtain approximate periodic solutions to the nonlinear oscillator describing the retaining wall dynamics subject to swelling pressure. The equation of the nonlinear conservative oscillator was established by using the single-mode Galerkin approach. It was shown that the zero initial value problem for the mass lumped model has only periodic solutions. Various approximations for frequency and amplitude of the periodic oscillations were verified against the numerical solution. In our forthcoming work, the relevance of transverse deformations of the retaining wall and its resulting oscillations under the influence of seismic vibrations will be investigated.

## Data Availability

The datasets generated during and/or analyzed during the current study are available from the corresponding author upon reasonable request.

## Alphabets

*A*: Dimensionless maximum deflection (–)
${A}_{b}$: *sH* Area of cross section of wall (m^2^)
$$B$$: $$\frac{\mathrm{ln}\left(10\right)}{cd}$$ (1/m)
$c$: 0.03 Empirically found swelling parameter (–)
*d*: Thickness of clay layer (m)
*E*: Modulus of elasticity of wall material (Pa)
$H$: Depth of wall (m)
$I$: $\frac{s{H}^{3}}{12}$ Moment of inertia of wall (m^4^)
*L*: Height of wall (m)
$s$: Width of wall (m)
*X*: Dimensionless deflection of wall (–)

## Greek symbols

${\mu }_{1}$: 1.8751040687 First positive root of the transcendental equation $1+\mathrm{cosh}\left(\mu \right)\mathrm{cos}\left(\mu \right)=0$ (–)
$\rho$: Density of wall material (kg/m^3^)
${\upsigma }_{0}$: Maximum swelling pressure (Pa)
*ω*: Dimensionless angular frequency (–)
